# Vascular pythiosis caused by *Pythium aphanidermatum*: the first case report in Asia

**DOI:** 10.1186/s40001-021-00603-w

**Published:** 2021-11-14

**Authors:** Pannaporn Thongsuk, Rongpong Plongla, Arsa Thammahong, Jaruwan Tiewsurin, Navaporn Worasilchai, Ariya Chindamporn, Chusana Suankratay

**Affiliations:** 1 Division of Infectious Diseases, Department of Medicine, Faculty of Medicine, Chulalongkorn University, Bangkok, 10330 Thailand; 2 Department of Microbiology, Faculty of Medicine, Chulalongkorn University, Bangkok, 10330 Thailand; 3 Division of Infectious Diseases, Department of Medicine, Buddhachinaraj Hospital, Phitsanulok, 65000 Thailand

**Keywords:** Pythiosis, *Pythium*, *Pythium insidiosum*, *Pythium aphanidermatum*, Case report, Asia

## Abstract

**Background:**

*Pythium*, soil-borne plant pathogens, are in the class Oomycetes. They are not true fungi, but are related to diatom and algae. There are two human pathogens including *P. insidiosum* and *P. aphanidermatum*. To date, only one case of pythiosis caused by *P. aphanidermatum* has been reported. We present herein the first case of *P. aphanidermatum* vascular pythiosis in Asia.

**Case presentation:**

A 47-year-old Thai woman, living in North Thailand, with ß thalassemia/hemoglobin E presented with acute recurrent arterial insufficiency of both legs. Emergent embolectomy with clot removal was performed. The pathology of the clot exhibited noncaseous granulomatous inflammation with many fungal hyphal elements. PCR identified *P. aphanidermatum* with 100% identity. Final diagnosis is vascular pythiosis. Unfortunately, the patient eventually expired after treatment with itraconazole, terbinafine, azithromycin, and doxycycline.

**Conclusions:**

To date, only one case of pythiosis caused by *P. aphanidermatum* has been reported. We present herein the first case of *P. aphanidermatum* vascular pythiosis in Asia.

## Background

*Pythium* are soil-borne plant pathogens in swampy areas in Thailand and many tropical and subtropical countries [1]. Based on the phylogeny, they are more related to diatom and algae than fungi. They belong to the family Pythiaceae, order Pythiales, class Oomycetes, phylum Oomycota, and kingdom Straminipila [1]. Pythiosis is an emerging, life-threatening infectious disease in humans [2–5]. *Pythium* have two forms including perpendicular branching hyphae and biflagellate zoospore [6]. The zoospore plays a major role in the pathogenesis in humans; it swims to attach and invade the host tissue [6, 7]. To date, there has been the largest case series of pythiosis reported from Thailand [5], however, the disease was reported from Australia, Asia, and America [3]. Most of the patients are farmers with predisposing thalassemia and other hemoglobinopathies. There are four categories of clinical manifestations including: (1) vascular (most of cases), (2) ocular, and (3) skin and subcutaneous, and disseminated pythiosis [2, 5].

There are two human pathogens including *P. insidiosum* and *P*. *aphanidermatum*. To date, only one case of pythiosis caused by *P. aphanidermatum* has been reported [8]. We present herein the first case of vascular pythiosis caused by *P. aphanidermatum* in Asia.

## Case presentation

**Fig. 1:**
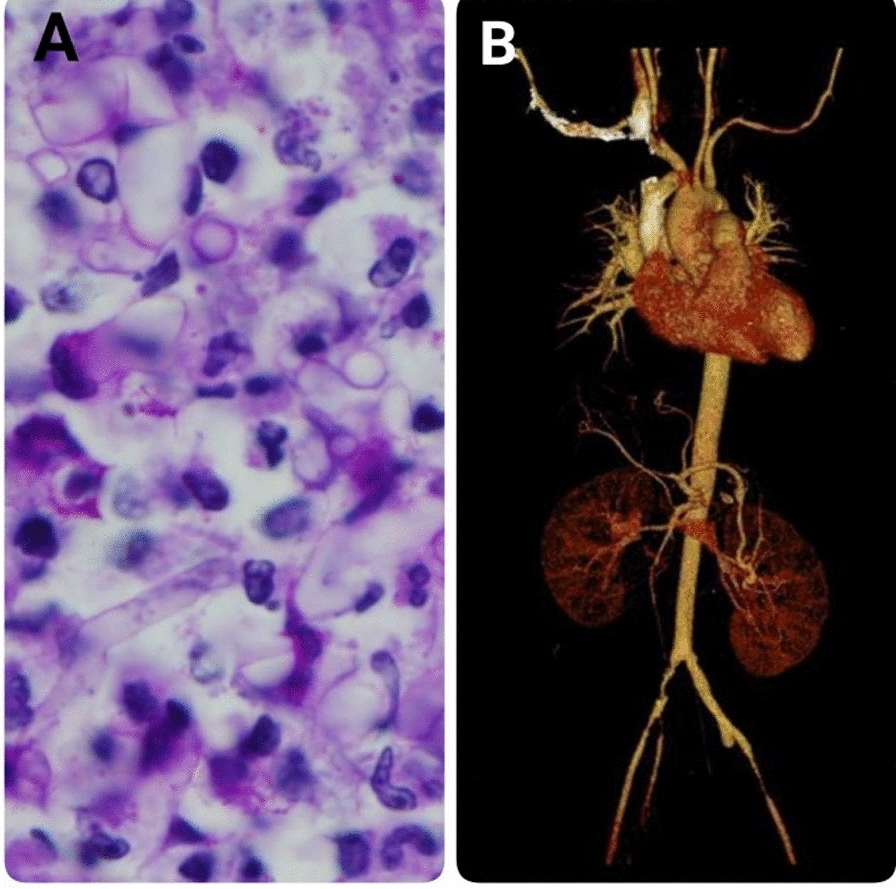
**A** Hematoxylin and eosin stain of the clot showing many fungal hyphae. **B** Computed tomogram angiogram showing near total occlusion of bilateral internal iliac arteries and narrowing of distal aorta and bilateral common iliac arteries

A 47-year-old Thai woman, living at Maesot, Tak, North Thailand, with ß thalassemia/hemoglobin E, was referred from a provincial hospital for further investigations regarding acute arterial insufficiency of both legs. Two months prior to admission (PTA), she noted a self-limited blackish painful nodule at left labia minor. Few weeks later, she developed a low-grade fever with bilateral groin pain. One month PTA, she had severe pain at left foot, and a diagnosis of acute arterial insufficiency was made a doctor at provincial hospital. Emergent embolectomy with clot removal at left common iliac artery was performed. Few days after hospitalization, there was a recurrent limb ischemia, and embolectomy was performed again. One week after hospitalization, the pathology of the clot exhibited noncaseous granulomatous inflammation with many fungal hyphal elements (Fig. 1A), and hence the patient was referred to King Chulalongkorn Memorial Hospital (KCMH), Bangkok, Thailand, for further investigations. She did not smoke. Unfortunately, the operation could not be performed due to the computed tomogram angiogram showing circumferential soft plaques along distal aorta, bilateral common iliac arteries, and external iliac arteries as well as near total occlusion of bilateral internal iliac arteries (Fig. 1B). Serum IgG titers against *P. insidiosum* was1:800 (in-house enzyme-linked immunosorbent assay, KCMH), and serum ß-d-glucan was 523 pg/mL. Unfortunately, the organism could not be isolated from the clinical specimens. The definite identification of the organism from the clot, by amplifying the internal transcribed spacer (ITS) of ribosomal DNA using the polymerase chain reaction (PCR) technique with sequencing of the amplicon and GenBankBLAST searching as previously described [9], was *P. aphanidermatum* (100% identity). Unfortunately, the patient eventually expired due to uncontrolled sepsis 2 weeks after treatment with itraconazole, terbinafine, azithromycin, and doxycycline as well as an iron chelator, deferoxamine.

## Discussion

The first case of pythiosis caused by *P. aphanidermatum* infection was described by Calvano and colleagues in the year 2011. The patient was a 21-year-old Hispanic male soldier who had extensive wound infection affecting both legs, right arm, and buttock in an explosive device blast injury in Afghanistan [8]. The patient underwent multiple operations for tissue debridements of legs without improvement, finally needing bilateral hip disarticulation. He expired 16 weeks after his final operation despite antifungal treatment with liposomal amphotericin B and voriconazole. Pre- and post-disarticulation fungal cultures of the necrotic tissues from both legs recovered *Mucor circinelloides*, *Aspergillus flavus*, and *P. aphanidermatum*. In the authors’ opinion, it was not clear whether the infection caused by *P. aphanidermatum* was a coinfection or not. Our case had vascular pythiosis without known history of trauma, similar to those caused by *P. insidiosum* in most studies.

To date, there have been two genera of the class Oomycetes causing human diseases including *Pythium* and *Lagenedium* [2, 10]. *Lagenedium giganteum* was reported to cause keratitis mimicking ocular pythiosis caused by *P. insidiosum* [10]. Of the genus *Pythium*, there have been two species of human pathogens including *P. insidiosum* and *P. aphanidermatum*. *P. aphanidermatum* is also a plant pathogen [11]. The biologic behavior as well as the human diseases caused by this organism, a member of the class Oomycetes, is similar to those caused by *P. insidiosum*. In addition, the morphology from the pathology of clinical specimens could not be distinguished between the two species. Both of them are irregularly branching, pauciseptate hyphae present within the arterial walls [8]. Hence, the differentiation between the two species requires the molecular technique using the PCR technique with primers specific for ITS region of ribosomal DNA. In our case, we previously thought that her vascular pythiosis was unquestionably caused by *P. insidiosum*. Surprisingly, it turned out to be *P. aphanidermatum*. We believe that vascular pythiosis can be caused by either *P. insidiosum* or *P. aphanidermatum*, and the latter may be underestimated due to similar clinical manifestations, morphology, and false-positive serum antibody using *P. insidiosum* enzyme-linked immunosorbent assay, as in our case.

Of the most reported cases of vascular pythiosis, the skin at the foot is the most entry site of infection [2, 5]. After the inoculation, the organism is angiotropic to the arterial wall, usually the dorsalis pedis or posterior tibial artery. The organism will slowly ascend via arterial wall to the distal aorta, and cause occlusion from a thrombus and/or fibrosis, resulting in arterial insufficiency of the leg. Our patient presented with a blackish painful nodule at genitalia which is likely the entry site of infection. And then the organism ascended bilaterally along the arterial wall to the internal iliac arteries, common iliac arteries, and finally the distal aorta. The postulation is confirmed by the findings from computed tomogram angiogram which demonstrated the near total occlusion of internal and common iliac arteries as well as distal aorta, with preserved arteries of both legs.

Due to no effective antimicrobials against *Pythium*, the surgery is the main choice of treatment of vascular pythiosis, usually amputation of the involved limb with organism-free surgical margin by microscopic demonstration [2, 5]. In our patient, the radical surgery could not be performed, and immunotherapy with vaccine was not available. Hence, itraconazole and terbinafine with adjunctive therapy with azithromycin and doxycycline were given to the patient. Susaengrat and colleagues recently published two vascular pythiosis cases for whom radical surgery could not be performed, who were successfully treated with adjunctive azithromycin and doxycycline. [12].

## Conclusions

To date, only one case of pythiosis caused by *P. aphanidermatum* has been reported. We present herein the first case of *P. aphanidermatum* vascular pythiosis in Asia. There are no differences between pythiosis caused by *P. aphanidermatum* and *P. insidiosum* regarding the clinical manifestations, the predisposing conditions, and cross-reaction of serum antibody with our in-house enzyme-linked immunosorbent assay against *P. insidiosum.*

## Data Availability

Not applicable.
